# Effect of Running Exercise on Oxidative Stress Biomarkers: A Systematic Review

**DOI:** 10.3389/fphys.2020.610112

**Published:** 2021-01-20

**Authors:** Anand Thirupathi, Ricardo A. Pinho, Ukadike C. Ugbolue, Yuhuan He, Yao Meng, Yaodong Gu

**Affiliations:** ^1^Faculty of Sports Science, Ningbo University, Ningbo, China; ^2^Laboratory of Exercise Biochemistry in Health, Graduate Program in Health Sciences, School of Medicine, Pontifícia Universidade Católica do Paraná, Curitiba, Brazil; ^3^School of Health and Life Sciences, University of the West of Scotland, Scotland, United Kingdom; ^4^Faculty of Engineering, University of Szeged, Szeged, Hungary

**Keywords:** running, marathon, ROS, biomarkers, oxidative stress, antioxidants

## Abstract

**Background:** Exercise induced health benefits are limited by the overaccumulation of reactive oxygen species (ROS). ROS and further oxidative stress could potentially induce muscle damage which could result in poor exercise performance. However, predicting ROS induced oxidative stress in response to endurance training has several limitations in terms of selecting biomarkers that are used to measure oxidative stress.

**Objective:** The purpose of this study was to systematically investigate the suitable biomarkers that predict oxidative stress status among runners.

**Methods:** According to the Preferred Reporting Items for Systematic Reviews and Meta-Analyses (PRISMA) statement, a search for relevant articles was carried out on PubMed/Medline, ISI Web of Science, and Google Scholar using related search terms such as oxidative damage, ROS, exercise, physical training, running, marathon, and ultramarathon.

**Results:** Outcomes included (1) running programs like a half-marathon, ultramarathon, and iron-man race, (2) measuring biochemical assessment of oxidative damage markers such as malondialdehyde (MDA), protein carbonyl (PC), total antioxidant capacity (TAC), thiobarbituric acid reactive substances (TBARS), 8-Oxo-2'-deoxyguanosine (8-OH-dG), 4-hydroxynonenal (HNE), and F1-isoprostones, and enzymatic and non-enzymatic antioxidants level.

**Conclusions:** This study concluded that a running exercise does not elicit a response to specific biomarkers of oxidative stress, instead, oxidative damage markers of lipids, proteins, and various enzymatic and non-enzymatic antioxidants are expressed according to the training status of the individual.

## Introduction

Exercise induced health benefits are well-known realities that have positively impacted peoples' lives in terms of reducing or preventing non-communicable diseases such as obesity, cardiovascular diseases, and other chronic health problems (Knez et al., 2006). Due to easy accessibility, many people start as recreational runners with a goal to improve health and exercise benefits geared toward disease prevention and incremental progress in physical fitness. However, moderate to extreme running may damage cellular structure by inducing reactive oxygen species (ROS) production (Parker et al., 2018). The free radical theory of aging was originally described in the 1950s by Denham Harman (Harman, 1956). After that, several reports on the harmful effects of ROS have been continuously described (Schieber and Chandel, 2014; Pizzino et al., 2017). It is now known that ROS play a vital role in cellular function in terms of acting as a signaling molecule. Despite this beneficial role, it is crucial to balance or scavenge the ROS; otherwise, it could lead to damage within the cellular structure. Nonetheless, contraction-induced ROS generation has been shown to play an important physiological function in the regulation of both muscle force production and contraction induced adaptive responses of muscle fibers to exercise training (Powers et al., 2011).

Extreme training such as ultra-marathon running may result in a 10–20-fold increase in oxygen consumption that can inevitably always produce ROS. However, the level of ROS is the important factor which determines whether it is a friend or foe within the cell (Powers et al., 2020). Moderate exercise can increase the antioxidant's level which facilitates an optimal level of ROS, whereas high intensity exercise can induce ROS formation, giving the maximum cellular adaptation (Powers and Jackson, 2008). Additionally, assessing the oxidative damage is unequivocal during exercise because oxidative damage is varied according to the intensity and duration. This can ultimately bring into question the exact role of ROS and exercise performance (Knechtle and Nikolaidis, 2018). ROS can induce several adaptive signaling pathways in the skeletal muscle (Powers and Jackson, 2008). However, the mechanism by which it can induce those pathways that signal for improved exercise performance is poorly understood. Furthermore, the ROS steady state level may significantly contribute to such an effect instead of an elevated level of ROS. Steady-state concentrations of ROS are well-balanced by several enzymatic regulations. For example, superoxide dismutase (SOD) lowers the steady state level of superoxide and decreases the rate of H2O2 production (Liochev and Fridovich, 1991, 1994). Further, this can maintain the activities of catalase and peroxidase. These studies exposed the fact that superoxide radicals inactivated the catalase and peroxidase, and SOD is the reason for this. In the exercise condition, steady state and ROS levels are determined by both the rate of ROS production and the rate of ROS scavenging. Thus, a steady state in an exercise condition can display either an overall increase or decrease in ROS formation in the entire human body, and exercise induced ROS formation is counterbalanced by their elimination and /or the prevention of formation of ROS which in concert can typically maintain a steady-state ROS level. Oxidative stress is not only a phenomenon that refers to elevated levels of ROS that damage lipids, proteins, and DNA, but it also plays a significant role in physiological changes through the interaction with cysteine (Cys) residues of proteins. For example, H2O2 interacts with Cys thiolate anions (Cys-S^−^) at physiological pH and oxidizes them to their sulfenic form, causing structural changes within the target protein and altering its function. This scenario drives alteration in the protein function that affects the transcription, phosphorylation, and other important signaling, and/or alter metabolic fluxes and reactions in the cells by altering enzymatic properties (Thirupathi et al., 2020). Therefore, it is important to consider the measurement of oxidative stress before it causes damage to the cells by affecting several physiological functions. However, measurement of oxidative stress in the cells has several limitations in terms of biomarker selection. This should run down the exact status of oxidative stress. Therefore, focusing on the underlying mechanism of adaptive signaling induced by ROS and selecting suitable biomarkers may facilitate runners that compete in long distance running by preventing ROS-induced damages in the skeletal muscles.

Running in events like a marathon or ultra-marathon can result in muscle injury, and the main factors that induce muscle injury are the activation of inflammatory cascades and oxidative stress, but measurement of oxidative stress has no particular suitable biomarkers as stated above (Niemelä et al., 2016; Knechtle and Nikolaidis, 2018). Therefore, this kind of sport may be a useful platform to find applicable biomarkers that can exactly predict the oxidative stress status in the cells. Moreover, there have been several arguments on whether extreme training sessions like ultra-marathons may increase the health benefits of physical exercise. The level of oxidation response (ROS level) which improves the exercise performance or increases the exercise-induced benefits is ambiguous (Mrakic-Sposta et al., 2020). Measuring the oxidative damage by selecting suitable biomarkers, nutrition, individual physical condition, type, and intensity of running exercise among the runners (Mrakic-Sposta et al., 2020) are all aspects that should be focused on. However, no studies have firmly established these aspects in terms of improving running exercise performance and the benefits. Therefore, the aim of this study was to present a systematic overview of published articles and to find the suitable biomarkers that predict oxidative stress among long-distance runners.

## Methods

### Search Strategy

In accordance with guidelines for the Preferred Reporting Items for Systematic Reviews and Meta-Analyses (PRISMA) statement, a search for relevant articles was carried out on PubMed/Medline, ISI, Web of Science, and Google Scholar using a broad range of synonyms and related search terms namely running, marathon, ultramarathon, oxidative damage, biomarkers, ROS, and exercise in works published between 1995 and 2020. To avoid the risk of missing relevant articles, additional papers were searched on the gray literature (i.e., generic Web search) and through the bibliography of previous reviews. One author (AT) ran the search and screened the initial titles after duplicates were removed. Two authors (AT and GY) independently examined potentially relevant articles in depth. We included only papers published in peer-reviewed journals which reported findings from experimental controlled studies, i.e., human studies only. We excluded articles not available in English, unpublished papers, and conference posters, or those reporting findings of non-experimental studies (e.g., pre-/post-intervention studies, case series, etc.). First author's name, year of publication, sample of intervention and control group, design and duration of the study, topic, type of intervention, outcome, assessment, and results were recorded using an electronic spreadsheet.

### Eligibility Criteria

#### Abstract Selection

The initial abstracts were searched through PubMed/Medline, Science Direct, and Google Scholar using the following criteria: (I) studies reported on participants in running programs that involved long distances between half-marathons and marathons, i.e., we included studies where the participants ran more than 10 km per race. (II) The runners had to be competitive, and participants that required medical support were omitted. (III) Search outputs included only articles that were peer-reviewed and published in English language journals. (IV) Only running programs like half marathons, marathons, and ironman races were included as types of interventions. (V). Only parameters that were related to oxidative damage and some studies on inflammatory responses that induce oxidative stress were included as types of outcome measures.

### Full-Text Articles Selection

The abstracts of the articles were further narrowed down using the following criteria: *Inclusion criteria:* We included prospective cohort studies, cross sectional studies, and randomized clinical trials. *Exclusion criteria:* We excluded different sport activities other than running programs.

### Risk of Bias Assessment

The risk of bias assessment was performed independently by two authors based on the Cochrane Risk of Bias Assessment Tool. A third author was consulted in case of any disagreements.

### Data Extraction and Analysis

For each study, the study characteristics (e.g., authors, nationality of the first author and published year), participant characteristics (e.g., number of participants, age, and gender), and the interventions (e.g., type, intensity, and running duration) were extracted and identified by an author and verified by another author based on running-induced changes on the biochemical parameters of oxidative stress and inflammation—which is related to inducing oxidative stress. All the parameters were evaluated in blood samples collected during or after the running program. Disagreements were resolved through discussions with other authors.

## Results

### Search Results

**Figure 1:**
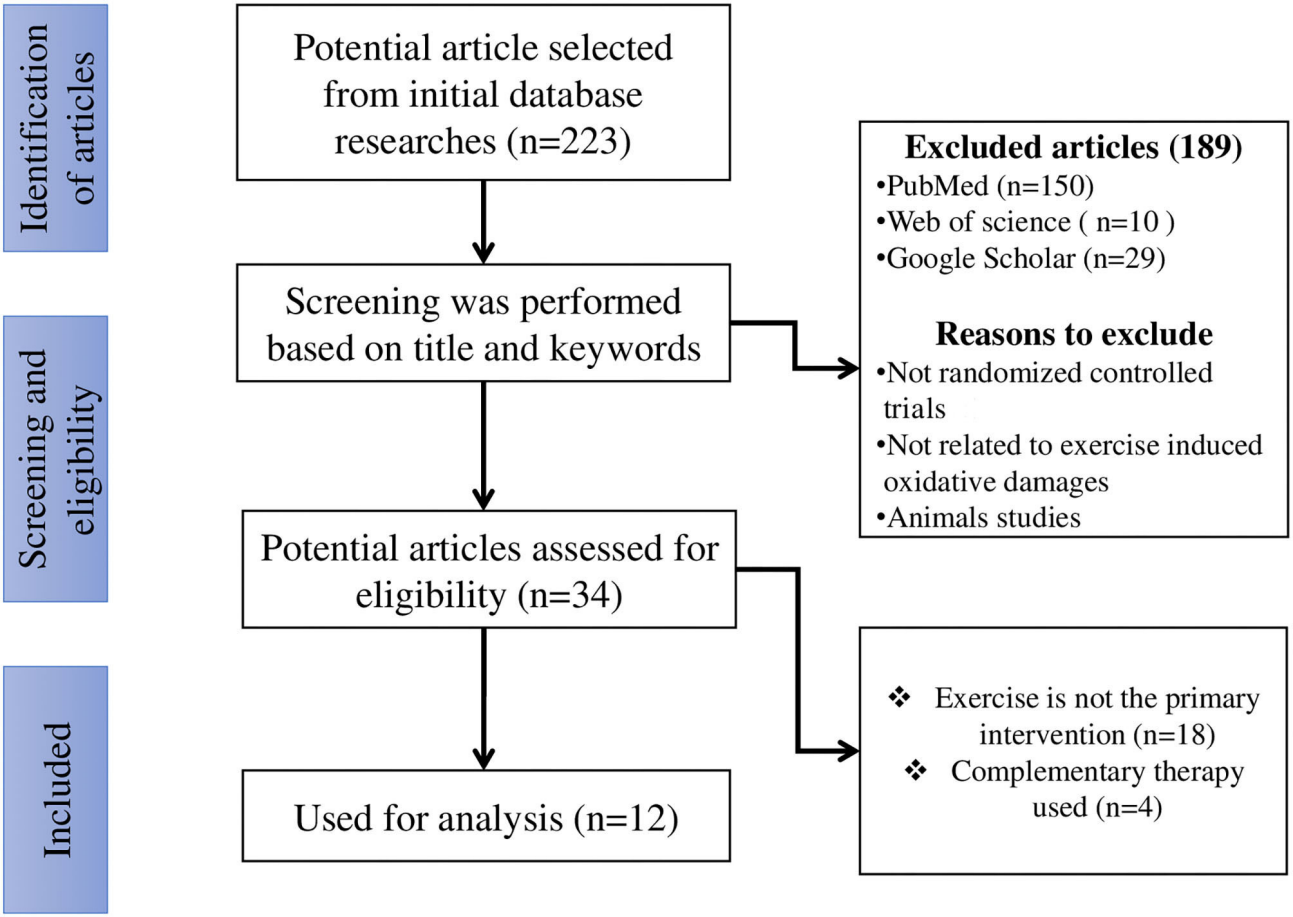
The search flowchart for screening process.

After evaluating 813 titles and abstracts, 223 articles were identified as potentially relevant from initial data base searches (Figure 1). After screening was performed using titles and relative keywords, 189 articles were excluded. The remaining 34 potential articles (full texts) were carefully evaluated, and 22 articles were excluded. The full texts of the remaining 12 articles were retrieved and reviewed, which were then included for systematic analysis.

### Participants Characteristics

A total of 12 studies were included in this study. Study population, the number of participants, mean age and SD, intervention, and main outcomes are outlined in Table 1.

**Table 1:** The study characteristics of included studies.

**Authors**	**Study population**	**Age**	**No. of participant**	**Intervention**	**Sample**	**Biomarkers used**	**Main results**
Vezzoli et al. (2016)	Italy	41.8 ± 5.98	30	All the participants took part in a double ring 50 km-long competitive ultramarathon race, performed during two consecutive editions.	Plasma/Urine	ROS↑ PC↑ TBARS↑ TAC↑ 8-OH-dG↑	The study results observed that PC, TBARS, and 8-OH-dG production were significantly increased, but it is directly related to the ROS production rates, suggesting that prolonged ultra-endurance induces ROS production and oxidative stress, but it is directly related to exercise duration.
Mrakic-Sposta et al. (2015)	Italy	45.0 ± 8.75	46	The study was carried out during two consecutive editions of extreme ultra-marathon for 330 km, an altitude difference of 24,000 m. The maximum time for completing the race was 150 h, and the distance was divided into seven stages with 35 relax stations.	Blood/Urine	8-OH-dG ↑ 8-iso PGF2α↑ Antioxidant capacity ↓	Exhaustive and prolonged exercise promotes ROS and oxidative stress.
Spanidis et al. (2017)	Greece	46/65	12	The participants performed at one of the most extreme mountain ultra-marathons. The distance was 103 km and the maximum time for race completion was 28 h.	Plasma/erythrocyte	TBARS ↔ PC ↔ TAC ↔ GSH↓ CAT ↔ sORP ↑	GSH level was increased postrace whereas PC, TBARS, TAC, and CAT were not significantly altered in postrace.
Pinho et al. (2010)	Brazil	34.7± 1.15	27	The participants involved performed a 3.8-km swim, followed by a 180 km bike ride, and finished with a 42.2 km marathon run	Blood	TBARS↑ LHP ↑ PC ↑ SOD ↑ CAT ↑	TBARS and PC were increased in postrace, so also were SOD and CAT levels suggesting that this triathlon induced oxidative damage and antioxidant enzymes.
Mastaloudis et al. (2001)	USA	45.0± 3.00	11	Participants ran a 50 km ultramarathon, and all the participants completed the race.	Plasma	F2-isoprostane↑ Alpha-tocopherol ↑ ascorbic acid ↑	Endurance exercise increased the F2-isoprostanes during 50 km ultramarathon along with increasing ascorbic acid and alpha-tocopherol which may suggest enhanced antioxidant defense in response to oxidative stress.
Radák et al. (2000)	Hungary	26.0–45.00	5	The runners participated in a marathon race over the following distances 93 km, 120 km, 56 km, 59 km, respectively.	Serum/ Urine	8-OH-dG ↓ CK ↓	This study showed the increased oxidative damage after running 328 km during the 4-day competition; however, oxidative damage did not increase further, suggesting extreme exercise cause adaptation that normalizes oxidative DNA damage
Liu et al. (1999)	Finland	31.0± 3.00	10/11	The subjects ran a full marathon (42.2km) and the running time varied from 3,13 to 5.52 h.	Plasma	TRAP ↑ α-tocopherol↔ β-carotene ↔ retinol ↔	Single bout of heavy exercise increased the TRAP and some of its components like uric acid, but it could be an adaptive defense mechanism against oxidative stress
Wagner et al. (2010)	Austria	35.3± 7.00	48	Participants were performed racing which was consisted of 3.8 km of swimming, followed by 180 km cycling and 42.2 km running.	Plasma	MDA ↑ CD ↑ OxLDL ↑ α and γ tocopherol ↑ β-carotene ↑	Single bout of ultra-endurance exercise is associated with elevated level of oxidative stress and the imbalance of oxidant/antioxidant sustained for 1 day after the exercise, the chronic exercise induced the biochemical adaptations against oxidative stress
Hessel et al. (2000)	Germany	41.6± 6.70	18	Healthy trained long-distance runners who attended the Berlin Marathon were included in this study.	Blood/ Plasma/ erythrocyte	Lipid peroxide ↑ GSH/GSSG↑ SOD↓ GPx ↓	The level of lipid peroxides and GSSG increased and SOD and GSH-Px activity was decreased
Suzuki et al. (2003)	Japan	31.7± 5.00	10	The participants ran for 42.195 km at 4 km intervals on the running program.	Serum/ Plasma	CK ↑ Uric acid ↑ IL-6 ↑ IL-8 ↑ IL-10 ↑ MPO ↑ TNF- α↓	Anti-inflammatory cytokines such as IL-6, IL-8, IL-10, and plasma free radical scavenging activity were increased after the race
Larsen et al. (2020)	Denmark	29.0-60.00	20	The running program was conducted in relation to Copenhagen Marathon 2018	Urine	8-oxodG↓ 8-oxoGuo↓	Oxidatively generated DNA and RNA were unaffected after running, but they were decreased after 4 days of running suggesting adaptive antioxidant effects.
Nieman et al. (2003)	USA	65.0–69.00	45	Participants ran 160 km under a condition that the runners had to complete the race within 30h.	Plasma	F2-isoprostane↑ Lipid hydroperoxide↑ FRAP↑	Runners competing in the 160 km had large perturbations in the oxidative markers of blood.

*ROS, Reactive oxygen species; PC, protein carbonyl; MDA, malonaldehyde; TAC, total antioxidant capacity; SOD, superoxide dismutase; GPx, glutathione peroxidase; CK, Creatine kinase; IL- 6, interleukin−6; IL- 8, interleukin−8; MPO, myeloperoxidase; TNF- α, tumor necrosis factor alpha; CAT, catalase; GSH, glutathione;TBARS, thiobarbituric acid; 4-HNE, 4-hydroxynonenal; LHP, lipid hydroperoxide; 8-oxodG, 8-hydroxydeoxyguanosine; 8-oxo-dG, 8-oxo-2'-deoxyguanosine; FRAP, Ferric reducing ability of plasma*.

### Study Selection

This study selected 12 articles to assess the effect of running exercise protocols on oxidative stress parameters. Fourteen articles were identified by searching databases and two were identified by the article's reference for inclusion in the analysis. All the records used in this study were based on human subjects.

### Risk of Bias of Included Studies

**Figure 2:**
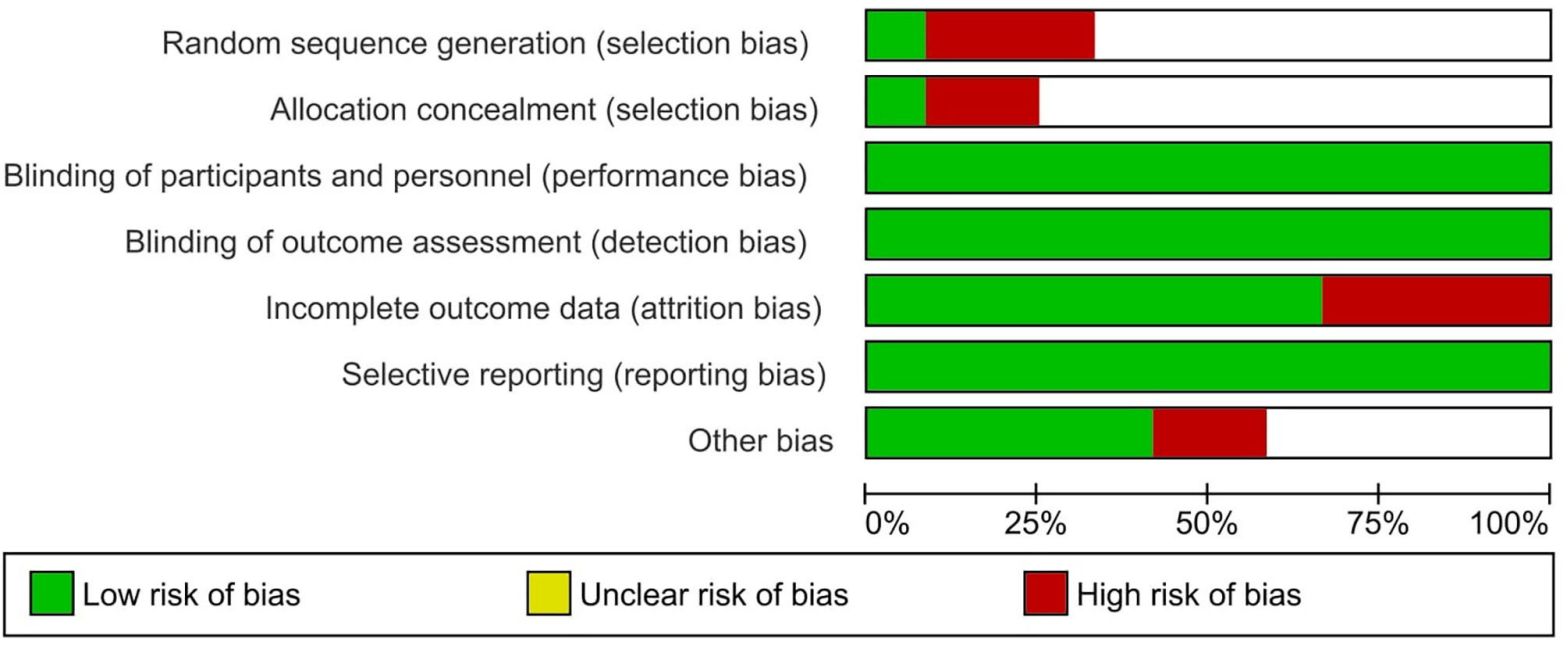
Risk of bias evaluation of included studies.

From the 12 included studies, at least six studies had risk of bias. Three studies had high risk in random sequence generation and allocation concealment. Four studies had a high risk in incomplete outcome data and two studies had high risk in other biases. Six studies had unclear risk in randomization and allocation concealment. Three studies had low risk in randomization and allocation concealment. Eleven studies had low risk in blinding of participants, and four studies had high risk in blinding of outcome assessment. All the studies had low risk in selective reporting (Table 2). Four studies had low risk in other biases and six studies had unclear risk in other biases (Figure 2).

**Table 2:** Risk of bias evaluation of included studies.

**Trials**	**Random sequence generation**	**Allocation concealment**	**Blinding of participants and personnel**	**Blinding of outcome assessment**	**Incomplete outcome data**	**Selective reporting**	**Other Bias**
Vezzoli et al. (2016)	High	Unclear	Low	Low	High	Low	High
Mrakic-Sposta et al. (2015)	Unclear	Unclear	Low	Low	Low	Low	Unclear
Spanidis et al. (2017)	Unclear	Unclear	Low	Low	High	Low	Unclear
Pinho et al. (2010)	Unclear	Unclear	Low	Low	Low	Low	Low
Mastaloudis et al. (2001)	Unclear	Unclear	Low	Low	Low	Low	Unclear
Radák et al. (2000)	High	High	Low	Low	Low	Low	Low
Liu et al. (1999)	Low	Low	High	High	Low	Low	Low
Wagner et al. (2010)	Unclear	Unclear	Low	Low	High	Low	Unclear
Hessel et al. (2000)	Low	Low	Low	Low	Low	Low	Low
Suzuki et al. (2003)	Unclear	Unclear	Low	Low	Low	Low	High
Larsen et al. (2020)	High	High	Low	Low	High	Low	Unclear
Nieman et al. (2003)	Low	Low	Low	Low	Low	Low	Unclear

### Effect of Exercise on Oxidative Stress Markers

After the first study that suggests exercise increases oxidative stress by Dillard et al. in 1978, a plethora of reports have shown that exercise increases oxidative stress in humans or animals. These studies mostly used cycle ergometer or treadmill exercises in which the participants used maximal or submaximal exercise in a climate-controlled laboratory. This compromises the prediction of the oxidative stress status among the exercised people. Therefore, to predict oxidative stress, it is important to assess suitable oxidative damage markers in various running platforms. One study showed neutrophilia and enhanced PMN capacity to generate oxygen radicals after running. This is the point where the oxygen radicals are established in the runner's blood and are evidenced by increased levels of LPO and GSSG as well as decreased level of SOD and GSH-Px (Hessel et al., 2000). Another study showed that a single bout of endurance exercise increases TRAP and some of its components like uric acid, but this was due to an adaptive mechanism against running-induced oxidative stress. The intense endurance exercise increased MDA which may react physiologically with several nucleosides to form adducts to deoxyguanosine and deoxyadenosine, and increased exercise intensity may increase the purine oxidation which results in an increase in the formation of uric acid (UA). This may be due to an adaptive mechanism against running-induced oxidative stress. Further, endurance training increases the high rate of ATP hydrolysis compared to its resynthesis which further stimulates the myokinase reaction and adenosine monophosphate deaminase reaction. Consequently, the adenine nucleotide pool decreased. Inosine-5'-monophosphate (IMP), hypoxanthine (Hx), xanthine (X), and UA are exercise related products of adenine nucleotide degradation that accumulate in the skeletal muscle or efflux into the blood which ultimately decreases the adenine nucleotide pool precursors (Zieliński et al., 2019). However, adenine nucleotide pool restoration may be slow and energy consuming, and *de novo* synthesis from the purine Hx is the only compound that may be reconverted and reutilized into the adenine nucleotide pool after being catalyzed by hypoxanthine-guanine phosphoribosyltransferase (HGPRT). Intense exercise increases the Plasma Hx significantly. Therefore, it is considered as an index of exercise intensity (Rychlewski et al., 1997). Furthermore, high intensity exercise limits the efflux of purines to the plasma resulting in reduced muscle nucleotide loss in active men (Hellsten-Westing et al., 1993b). Six weeks of high intensity exercise decreased the level of Hx both at rest and after the exercise, and this may be due to muscle adaptation that leads to a reduced adenine nucleotide (Hellsten-Westing et al., 1993a). Further, this study showed that a reduced level of thiol content was efficiently utilized by the ROS after the race (Liu et al., 1999). An additional study showed that prolonged ultra-endurance exercise causes an increase in ROS production and oxidative stress, but it is dependent on specific biomarkers and the exercise duration (Vezzoli et al., 2016). A different study investigated the effect of running on oxidative modification of nucleic acid, and it was found that marathon participation immediately induced an inflammatory response, but it did not increase the oxidative modification of nucleic acid, instead, it decreases the oxidatively generated nucleic acid modifications, suggesting an adaptive antioxidant effect following running (Radák et al., 2000; Larsen et al., 2020). One study showed that even after the running, the oxidative stress lasted for up to 3 days. Additionally, this study showed that capacity oxidation-reduction potential (cORP), and GSH are the most effective markers for analyzing running-induced oxidative stress (Spanidis et al., 2017). Two studies investigated the ironman triathlon's effect in inducing oxidative damage. From those two studies, one study showed that there is no persistent oxidative stress in response to an iron-man race (Wagner et al., 2010), whereas the other study showed that an ironman race provoked significant alterations in oxidative stress and inflammatory parameters (Pinho et al., 2010). Another study showed that increased oxidative stress regulates the inflammatory process during heavy exertion (Nieman et al., 2003; Suzuki et al., 2003). Another study showed that heavy endurance exercise increased the lipid peroxidation (Mastaloudis et al., 2001). One study showed that exhaustive and prolonged exercise induces oxidative stress and inflammation (Mrakic-Sposta et al., 2015).

## Discussion

This systematic review analyzed the effect of different running programs on oxidative stress with the aim of determining suitable biomarkers that predict the early oxidative stress status in runners. From the 12 selected and systematically reviewed articles, running exercises do not elicit a response to specific biomarkers of oxidative stress, instead, oxidative stress markers like ROS induced end products of lipids, proteins, and various enzymatic and non-enzymatic antioxidants expressed according to the training status of the individual.

### Different Markers on Oxidative Stress Measurement

Although it is known that exercises like running can induce oxidative stress, the methods that potentially measure the oxidative damage are limited because some of the methods have failed to reflect the exact status of oxidative stress in the cells. Consequently, the measurement of oxidative stress is required and is a more promising approach in different physiological conditions induced by exercise. Measurement of cellular ROS is one of the direct ways to determine the oxidative stress. For example, fluorogenic probes are used as a direct method to measure superoxide radicals, hydrogen peroxide, hydroxyl radicals, and peroxyl radicals (Debowska et al., 2015). Other ways to assess the oxidative stress include ROS derived metabolites (D-ROMS). However, these measurements are compromised in predicting its accuracy because the radicals that are assessed using direct measurements are relatively short lived and highly reactive (Denicola et al., 1998). Additionally, different ROS have different degrees of reactivity toward cellular components, and the free iron availability is considered crucial for ROS toxicity due to the role it plays in the Fenton reaction to produce hydroxyl radicals. Therefore, indirect measurement could be a useful platform to determine ROS induced oxidative stress. For example, ROS induced damage to lipids, proteins, and nucleic acids and its further end product assessment could be a promising approach to assess the oxidative stress in the samples of people that exercise. For example, all the studies that we selected with the aim of finding the suitable biomarkers, have assessed the ROS induced end products like PC, MDA, TBARS, 8-OH-dG, and F2-isoprostanes, but no studies firmly reported the suitable biomarkers to measure the oxidative damage because sample type, collection of sample timing, and exercise duration and type may frequently change the reaction time of the ROS, which may compromise the prediction of ROS induced oxidative stress. Further, measuring the level of antioxidant compounds such as enzymatic, non-enzymatic compounds, and some low molecular mass compounds are useful candidates for evaluating oxidative stress in the samples. However, frequent changes in ROS concentration due to duration, intensity, and type of exercise may mispredict the expression level of those enzymatic and non-enzymatic antioxidants. For example, one study reported that the GSH level increased after the race whereas the CAT level was not significantly increased (Spanidis et al., 2017). Another study reported that the CAT level increased after the race (Pinho et al., 2010). These contradicting results may be because the concentration of ROS differed in different running statuses such as in distance and the time in which the race was completed.

### Type of Exercise and Oxidative Damage Markers

Regarding exercise, different types of exercises influence the level of ROS induced end products based on the training status (Hadžović-Džuvo et al., 2014; Ammar et al., 2020). Furthermore, studies have shown that endurance exercise increased ROS and induced damage to lipids, proteins, DNA and antioxidant levels (Kanter et al., 1993; Niess et al., 1996; Michailidis et al., 2007). However, direct evidence on those oxidative damage markers is limited in reflecting oxidative stress, and some studies only observe a few markers that are increased during endurance training as well as some markers do not show signs of any increment (Alessio et al., 1997; Bloomer et al., 2006). Vezzoli et al. observed that prolonged ultra-endurance running increased the PC, TBARS, TAC, and 8-OH-dG (Vezzoli et al., 2016). Spanidis et al. reported that there were no changes observed during or after running in TBARS, PC, and TAC, suggesting that these outcomes are dependent on training status and specific biomarkers that are assessed during running (Spanidis et al., 2017). Further, this study reported that GSH and cORP are the most effective biomarkers to analyze running-induced oxidative stress. In addition, this study showed that these markers existed up to 3 days after the race, which is possibly due to the exercise intensity and total caloric expenditure. Indeed, several studies have shown that the oxidative stress response is altered in relation to exercise intensity (Alessio et al., 1988; Lamprecht et al., 2008). From these results, we conclude that assessing the oxidative damage markers in response to exercise running may vary according to exercise intensity, duration, and individual antioxidant capacity. No persistent results were observed in all the selected studies with regards to oxidative stress biomarkers. However, most of the studies used oxidative damage markers and individual antioxidant capacity such as PC, MDA, TBARS, CAT, and GSH for the measurement of oxidative stress, suggesting that assessing oxidative damage markers and individual antioxidant capacity could be a promising method to reflect the potentiality of methods on oxidative stress compared to the direct method that assesses the ROS.

### Inflammatory Markers on Oxidative Stress Measurement

The national institutes of health define the word biomarker as the process of both normal and abnormal processes in the biological system. Since there is no specific biomarker to predict the accurate status of oxidative stress, inflammatory markers could also be a useful candidate to assess the oxidative stress in exercise conditions. An exercise induced inflammatory response has long-term effects on human health, but ROS could be the driving factor for inflammation (Suzuki, 2018). ROS induce several signaling events that are directly involved in inducing inflammation during exercise, such as nuclear factor kappa-light-chain-enhancer of activated B cells (NFkB) and activator protein-1 (AP-1) (Biswas, 2016; Liu et al., 2017). Studies observed that running exercises increased the inflammatory response, but did not increase nucleic acid modifications by ROS, bringing into questioning the above statement of whether ROS could be a driving factor for inflammatory response or whether exercise-induced adaptive antioxidant effects could only detoxify the ROS without affecting inflammatory cascades (Radák et al., 2000; Larsen et al., 2020). However, one study reported that iron-man races increased the oxidative stress-induced inflammatory response (Pinho et al., 2010). In contrast, another study observed that no consistent changes were observed in oxidative stress parameters and inflammatory responses, suggesting that different exercise modalities have different effects on oxidative stress parameters and inflammatory responses (Wagner et al., 2010). For example, high-intensity prolonged running exercise induced the oxidative stress and inflammation, but even moderate continuous exercise increased the oxidative stress compared to discontinuous high-intensity exercise (Mastaloudis et al., 2001; Vezzoli et al., 2016). However, this moderate exercise-induced oxidative stress effect could be changed with duration. These varying results show the uncertainty of the argument that inflammatory markers cannot be used for assessing the oxidative stress. More research is therefore required to confirm the effect of inflammatory markers as an effective strategy to assess oxidative stress in exercise conditions.

### Effect of Exercise Intensity and Duration on Oxidative Stress

ROS generation depends on exercise intensity and duration, as exercise types differ in their energy requirements, level of oxygen consumption, and the mechanical stress imposed on tissues. During low-intensity and duration, protocols have effective antioxidant defense mechanisms that likely meet the ROS production, but, as the intensity and duration of exercise increases, the antioxidant defense is no longer adequate—potentially resulting in oxidative damage. A study has shown that neutrophil production of superoxide increased only at intensities above the lactate threshold in exercised men (Quindry et al., 2003). In contrast to the above study, other studies reported that oxidative stress markers in blood increased with 60-, or 120-min of exercise at a constant intensity. Several reviews conclude that regular exercise training does not lead to chronic oxidative stress in the active muscles which fosters the concept of exercise induced hormesis (Ji et al., 2016; Powers et al., 2020). Hormesis used to describe the biphasic dose response curve where small amounts of the stressor provide beneficial adaptive effects on cells, whereas high levels of those stressors may result in damage to the cells. From this, exercise induced low levels of ROS production play a crucial role in exercise induced adaptation of the skeletal muscle, and this can be explained using the bell shaped hormesis curve where the optimum level of ROS plays a role in muscle adaptation whereas when above the optimum level of ROS, it can lead to various damages to the cells and a decline in the exercise induced adaptation (Ji et al., 2016; Powers et al., 2020). These studies do not provide strong enough evidence to show that high intensity exercise for prolonged periods of time, can result in oxidant-mediated damage in the cells and decrease antioxidant capacity in the trained muscles (de Sousa et al., 2017; Radak et al., 2017; Di Meo et al., 2019). The reasons associated with this are the cardiovascular systems ability to affect the sustainability of high intensity by providing blood to the working muscles and affect the ROS production on muscle fatigue (Ji et al., 2016). Thus, the ROS production level is limited during exercise. Another reason is that mitochondrial coupling is higher in state 3 respiration during exercise resulting in the reduction of electron spill and ROS production by the mitochondria when compared to state 4 (resting) respiration. A final reason is that the exercise can increase the antioxidant enzymes in the skeletal muscle that supports the muscle fiber, to remove the ROS during exercise (Powers et al., 2020). These results predict that skeletal muscles are not exposed to ROS mediated damage during exercise.

### Nrf2 Signaling in Response to Exercise

Nuclear factor erythroid 2-related factor 2 (Nrf2) is a transcription factor that is considered to be a master regulator of the antioxidant defenses, facilitating more than 200 cytoprotective genes in response to oxidative stress (Tebay et al., 2015). Nrf2 is a family member of the basic leucine zipper that is repressed with Kelch-like ECH-associated protein 1 (Keap1) protein in a sequestering form in the cytoplasm under the unstressed condition. In response to oxidative stress, cysteine residues are modified on Keap1 which unhinge the Nrf2 from Keap1. Thus, Nrf2 is translocated into the nucleus where it can heterodimerize with small MAF proteins and bind to Cis-acting antioxidant response elements (AREs) which ultimately activate the enzymatic antioxidants. Exercise induced ROS formation may activate the Nrf2 which likely occurs through oxidation of cysteine residues as mentioned above. A single bout of exercise has been observed to increase the Nrf2 gene expression in wild type mice (Merry and Ristow, 2016). However, this study was performed using acute exercises that are not long enough to predict whether exercise can increase the Nrf2 gene expression or not. However, a recent study observed that acute exercise increased Nrf2 protein levels in the blood in young and older men (Done et al., 2016). Another study has shown that Nrf2 increased in moderate treadmill exercises (Scott et al., 2015), suggesting that fitness plays an important role in maintaining the nrf2 level. However, an increase of Nrf2 signaling is dependent on the duration of exercise (Done et al., 2016). For example, treadmill exercises at <1 h elicited no change in the Nrf2 level in the animals (Wang et al., 2016). In contrast, when the duration is increased, it is apparent that the Nrf2 level is increased in the skeletal muscle tissue (Li et al., 2015; Wang et al., 2016). Another animal study showed that 6 h of running clearly elicited an increased level of Nrf2, but no change occurred for 1 h of running (Li et al., 2015). In contrast to these studies, other studies observed that even 1 h of treadmill running increased the Nrf2 level (Merry and Ristow, 2016). The differences in these studies may be due to differences in the protocol intensities (Done et al., 2016). Recent work has reported that exercise mode, intensity, and duration can affect the Nrf2 cycling *in vivo* like influencing the frequency of the import/export of Nrf2 into and out of the nucleus (Lamprecht et al., 2008; Li et al., 2015; Xue et al., 2015). However, determining the optimal exercise dose or delivery on Nrf2 activation should be expanded on in future studies.

### Limitation and Future Direction

Although direct methods in assessing ROS could be a promising approach, as we mentioned earlier, the stability of the reactive molecules is short lived and highly reactive. Therefore, assessing these molecules in the biological system remains complicated. However, assessing the oxidative damage markers is one of the stable methods to provide more reliable results for the measurement of oxidative stress in the samples. Some complications still need to be eradicated such as assessing these oxidative damage markers that are only reflected to a local degree of oxidative stress, while others have a direct effect on target molecules. This further questions the applicability of those markers in assessing oxidative stress in the sample. Next, the sample collection should be processed with precaution to ensure the stability of the sample because there is a possibility for molecules to become more susceptible to be oxidatively damaging. However, non-invasive techniques could be useful to overcome normal sample collection procedures. For example, analysis of urinary biomarkers provides better applicability to measure oxidative damage because the sample collection is easy and has a low organic and metal content (Il'yasova et al., 2012; Marrocco et al., 2017). Additionally, a urinary sample minimizes the sample oxidation during sample collection and storage (Marrocco et al., 2017). Another advantage of a urinary sample is that it provides a longer period of the redox balance index when compared to blood. This can allow the urine sample to be more sensitive to predicting oxidative stress for longer periods. However, only a few markers have been validated in animals and humans, like F2-isoprostanes, 8-oxodG, and the MDA level detected by HPLC. Furthermore, some aspects like stability of the markers, particularly MDA and F2-isoprostanes variations, can produce esterifies lipids in the urine causing uncertainties in the applicability of these markers as effective methods for oxidative stress measurement. However, some promising markers like acrolein-lysine and dityrosine are understudied which could reflect the oxidative stress. This will diversify the current parameters in measuring oxidative stress in humans in the near future. As stated above, some inflammatory markers could be useful to measure the oxidative damage, but its specificity on local oxidative damage and target molecules is questionable because different physiological and pathological conditions induce different inflammatory cascades (Chen et al., 2017). Therefore, it cannot be recommended to measure oxidative damage as oxidative stress biomarkers. Regarding antioxidant status, everyone during exercise or before exercise have different antioxidant statuses to oxidative responses, which could provide conflicting results during antioxidant status measurement. For instance, some studies reported that exercise running increased the antioxidants (Mastaloudis et al., 2001), whereas other studies reported that these antioxidant levels are decreased for runners (Hessel et al., 2000). This could be due to an adaptive response that nullifies the ROS toxicities. To overcome these problems, it is suggested that determining total antioxidant status could be a useful parameter among runners.

Further, there is no specific biomarkers recommended for the measurement of oxidative stress for runners. However, it should be done based on assessing the training status of the individual. Therefore, an integrative approach is required for the measurement of oxidative stress before and after the exercise. A computed approach was recently used for the measurement of oxidative stress, such as OXY-SCORE or oxidative-INDEX, computed by subtracting antioxidative capacity from ROS levels/ROS induced damage or oxidative stress index (OSI), which is the ratio of total oxidant status to the total antioxidant status and can provide more insights for measuring the oxidative stress in the exercise condition. Finally, to the best our knowledge, there is no specific biomarkers or methodologies for the measurement of oxidative stress. More research to provide better and more reliable approaches to earlier prediction of oxidative stress in different types of exercise is therefore required. Further, before selecting an appropriate method to determine oxidative stress, a deep and critical analysis must be carried out according to the aim and design of the study, from the available literature, to select suitable biomarkers.

## Conclusions

This study potentially observed that different running programs at different intensities and durations induced oxidative damage, but better adaptive mechanisms in runners decreased the oxidative damage, suggesting that different modalities of running exercises have stronger effects on inducing oxidative damage, following adaptive mechanisms to counteract oxidative stress. However, this outcome is dependent on specific oxidative damage markers that are analyzed during the running program. Because some studies used direct methods to assess the oxidative stress, while other studies used oxidative damage markers as oxidative stress indicators, results to measure the exact status of the oxidative damage in the runners were conflicting. Furthermore, exercises like running can increase the level of antioxidants which reverse the oxidative damage. However, it should be noted that the selected studies had some methodological flaws and a high risk of bias justifying the effect of oxidative damage markers as an efficient method to assess the oxidative damage and running-induced adaptive response.

## Data Availability Statement

The original contributions presented in the study are included in the article/supplementary materials, further inquiries can be directed to the corresponding author/s.

## Author Contributions

AT, YH, and YM conceived the presented idea, developed the framework, and wrote the manuscript. AT, RP, UU, and YG provided critical feedback and contributed to the final version. All authors were involved in the final direction of the paper and contributed to the final version of the manuscript. All authors have read and agreed to the published version of the manuscript.

## Conflict of Interest

The authors declare that the research was conducted in the absence of any commercial or financial relationships that could be construed as a potential conflict of interest.
